# TB-related stigma is widely prevalent among people with TB and carers in Mongolia

**DOI:** 10.5588/ijtldopen.25.0174

**Published:** 2025-07-09

**Authors:** B. Tsogt, J.T. Denholm, N. Dambaa, T. Sambuu, N. Tsegeen, G. Munkhjargal, A. Chuluunbaatar, G. Dorj, G. Sukhbaatar, O. Adilaa, D. Dalai, O. Batmunkh, Z. Khukhkhuukhen, B. Ulziikhutag, Kh. Jargalsaikhan, N. Moyo, S.M. Graham

**Affiliations:** ^1^ Mongolian Anti-Tuberculosis Coalition, Ulaanbaatar, Mongolia;; ^2^ Victorian Tuberculosis Program, Melbourne Health, Melbourne, VIC, Australia;; ^3^ Department of Infectious Diseases, University of Melbourne, Parkville, VIC, Australia;; ^4^ National Center for Communicable Diseases of the Ministry of Health, Ulaanbaatar, Mongolia;; ^5^ National Center for Public Health of the Ministry of Health of Mongolia, Ulaanbaatar, Mongolia;; ^6^ National Human Rights Commission of Mongolia, Ulaanbaatar, Mongolia;; ^7^ The Global Fund Project Coordinating Unit, Ministry of Health of Mongolia, Ulaanbaatar, Mongolia;; ^8^ Country Coordinating Mechanism of the Global Fund supported TB and HIV projects, Ulaanbaatar, Mongolia;; ^9^ Melbourne Children’s Global Health, University of Melbourne Department of Paediatrics and Murdoch Children’s Research Institute, Royal Children’s Hospital, Parkville, VIC, Australia.

**Keywords:** tuberculosis, self-reported stigma, people with TB, healthcare workers

## Abstract

**BACKGROUND:**

In Mongolia, estimated TB incidence is high, but treatment coverage is low. Stigma is a likely barrier to access but has not previously been evaluated.

**METHODS:**

We conducted a national TB stigma assessment in Mongolia in 2021–2022 to evaluate prevalence and impact of TB-related stigma. All survey participants had face-to-face interviews conducted by trained community staff members using semi-structured questionnaires tailored to study participants.

**RESULTS:**

Study participants included 460 people with TB, 90 family members, 83 community members and 115 healthcare workers (HCWs). Overall, 53% of participants reported experiencing stigma with HCWs reporting highest prevalence (72%). Stigma was most commonly experienced in healthcare settings, particularly during initial diagnosis and treatment initiation. Urban slum residence was associated with higher stigma among patients while older age was associated with lower scores. Female respondents reported stigma significantly more commonly overall. Family members and community members commonly reported secondary stigma with concerns about infection and social isolation being prevalent.

**CONCLUSION:**

TB-related stigma is highly prevalent across Mongolia. There is an urgent need for comprehensive stigma-reduction strategies that extend beyond PWTB and their families to also include HCWs and broader community.

The WHO estimates that 2.7 million of the 10.8 million people who developed TB in 2023 were not treated.^1^ Mongolia is a WHO-listed high-burden country for TB and multidrug-resistant/rifampicin-resistant TB (MDR/RR-TB), with estimated annual TB incidence of 491 per 100,000 population. Although treatment outcome was successful for 87% of people treated for TB in 2023, treatment coverage was estimated to be only 18% compared to 75% globally.^1^ TB preventive treatment (TPT) coverage was also extremely low; 14% for household contacts. Understanding access barriers in Mongolia is critical for effective systemic responses. TB disproportionately affects marginalized groups in Mongolia. A National Prevalence Survey in 2014–2015 found 35% of participants were unemployed and living below subsistence, with a third belonging to vulnerable social categories.^2^ TB causes high catastrophic costs for the majority of TB affected families, further perpetuating vulnerability.^3^ TB incidence is particularly high among prisoners, institutionalized individuals with mental health disorders and homeless people, which may further intersect with discrimination and disadvantage.^4,5^ Discrimination is recognized as an important barrier, hindering care and adherence, and impacting overall TB control efforts.^6^

Understanding context-specific barriers to access and successful treatment completion in countries like Mongolia is critical for developing targeted and effective systemic responses. Research on the extent and systemic nature of discrimination and stigma is limited. We aimed to determine the prevalence of TB-related stigma and evaluate its impact, to inform and support the development of recommendations to address TB-related stigma.

## METHODS

### Study setting

Mongolia is a large land-locked country of 3.4 million people, and population density of only around two per square kilometre; approximately half live in urban settings.^7^ Estimated incidence of all TB and MDR/RR TB in 2023 is 491 and 34 per 100,000 population respectively with the highest number of cases in adult males (35–54 years).^1^ TB services are provided free of charge at state-run health facilities in Mongolia. Individuals presenting with TB symptoms at these health facilities are screened and, if found to have presumptive TB, are referred to centralised dispensaries located in every urban district and province, as well as to the National Center for Communicable Diseases, for diagnostic evaluation.^8^ However, TB services tend to be centralised and concentrated at provincial referral levels, especially services for MDR/RR-TB which are concentrated in Ulaanbaatar and 8 urban settings. In 2021, 559 healthcare workers (HCWs) provided facility-based services for TB and over 100 trained community HCWs provided community-based care.^9^

### Study design and population

We conducted a prospective mixed-methods study to evaluate and observe: the level and manifestations of anticipated stigma, self-stigma, and enacted (directly experienced) stigma in people diagnosed with TB; the settings and stages of care in which TB-related stigma is being experienced; the level and dimensions of secondary TB stigma experienced by family members or primary carers of people diagnosed with TB and stigma experienced by HCWs. The qualitative findings from interviews as well as on-site observational findings are reported separately. The assessment team and community stakeholders co-designed an evaluation protocol utilizing the Stop TB Partnership’s TB Stigma Assessment Tool and Implementation Handbook.^10,11^ To explore experiences of TB stigma, four distinct populations located in a wide range of settings across Mongolia were invited to participate:

1. People with TB (PWTB) who were adults (18 years and older) diagnosed with TB or with a history of TB treatment within the previous 12 months. A representative sample on basis of location (province/district; urban/rural) was selected randomly from all adults registered with drug-susceptible TB from September 1^st^ 2020 to September 30^th^ 2021 and listed in the electronic data system. No exclusions on the basis of disease site or treatment outcomes were applied. In addition, all 132 individuals treated for MDR/RR-TB from September 1^st^ 2020 to September 30^th^ 2021 were selected.
2. Family members or caregivers of enrolled PWTB.
3. Community members residing or working in the proximity of PWTB, including neighbours, schools, workplaces, and hospitals.
4. HCWs who provide care for PWTB: clinicians, nurses, and other mid-level specialists from each unit of the TB dispensary, along with doctors, nurses, and laboratory technicians from the National Centre for Communicable Diseases, were invited to participate in the study based on their availability.

A simple random sampling method was used to select PWTB from the national TB database with participants from all 21 provinces in Mongolia and all 9 districts in Ulaanbaatar. We calculated that a sample size of 480 people with TB would be required for our primary evaluation using the KNCV guidance^12^ and planned to additionally recruit up to 3 family members/caregivers per person, 3 community members per district and up to 3 HCWs per province and district. Details of the random sampling and sample size calculation are in the Supplementary Data. Supplementary Data Table S1 lists numbers of PWTB selected from each province and district. The characteristics of those selected were compared to the characteristics of all TB notifications and there were no statistically significant differences as regards age, sex, employment status, urban or rural residence, TB type (new or relapse) or site of TB (Supplementary Data Table S2). Participation was voluntary and anonymised, with written consent. All 4 groups of people who agreed to participate reported dichotomous outcomes regarding the presence of stigma and agreement with various statements about stigma experience. We administered five-point Likert-scale questions to further assess stigma in people with TB (12 questions) and HCWs (9 questions). Participants were instructed to choose only one option (from 0=strongly disagree to 4=strongly agree) per question. The data collection tools and consent form are in Supplementary Data.

### Data analysis

Self-reported stigma scores were assessed as a continuous variable using STATA v14.0 software.^13^ Total score could range from 0 to 48 for PWTB and 0 to 36 for HCWs, with a single response of 0-4 to each of 12 and 9 questions, respectively. For dichotomous responses to questions to all participants about stigma experience, descriptive analyses were used to describe the characteristics of participants and contexts as well as places where stigma was experienced. The stigma scores were specific to the group and so were only compared within and not between groups. Univariate linear regression analyses were conducted to assess associations between independent variables and stigma scores with all variables included in multivariable analysis. Logistic regression was also performed. Pearson’s chi-square test was used to determine the association between categorical variables.

### Ethics

The study received approval from the Scientific Committee of the National Center for Communicable Diseases and from the Medical Ethics Monitoring Committee of the Ministry of Health.

## RESULTS

Of 930 people invited to participate between 21 February to 2 April 2022, 748 (80.4%) agreed to participate in the study. Table 1 lists the numbers of participants and their characteristics with numbers and proportions of those who reported stigma overall and by participant age group and sex. Most participants were PWTB (n=460, 61.5%), either currently receiving treatment (n=124) or recently completed treatment (n=330); data of treatment unavailable for six participants. Of 448 whose treatment regimen was known, 59 were treated MDR/RR TB. Other study participants included family members (n=90, 12%), community members (n=83, 11%) and HCWs (n=115, 15.4%). Overall, 53% (397/748) of participants reported directly experiencing stigma in relation to TB (Table 1). Stigma was more commonly reported by HCWs (72%) compared to other sub-groups. Stigma was more commonly reported by people aged between 45 and 65 years of age, and significantly more commonly reported by women than men (59% v 46%; two-tailed p<0.001) with this difference widest in community members. The negative impact on seeking or having access to TB care was commonly reported by all groups. Perceived stigma was common among HCWs with 62% reporting experiencing stigma by other HCWs. Observed stigma was also common among these groups.

**Table 1.** Numbers and characteristics of those who experienced stigma by study participant categories.

Sub-groups of participants	People with TBN=460		Family memberN=90		Community members^A^N=83		Health care workersN=115		TotalN=748
% of participants	61.5%		12%		11%		15.5%		
Number (%) reporting stigma^λ^	215 (47%)		48 (53%)		51 (61%)		83 (72%)		397 (53%)
Characteristics: number (N) and number reporting stigma (NRS) (% of participants with characteristic within sub-group)									
*Age group*	N	NRS (%)	N	NRS (%)	N	NRS (%)	N	NRS (%)	NRS (%)
18-24 years N=73	65	31 (48%)	1	1 (100%)	4	3 (75%)	3	0 (0%)	35 (48%)
25-44 years N=375	241	118 (49%)	43	22 (51%)	34	19 (56%)	57	41 (72%)	200 (53%)
45-64 years N=256	128	58 (45%)	35	18 (51%)	38	26 (68%)	55	42 (76%)	144 (56%)
65 or older N=44	26	8 (31%)	11	7 (64%)	7	3 (43%)	0	0 (0%)	18 (41%)
*Sex*									
Female N=409	190	97 (51%)	67	36 (54%)	55	37 (67%)	97	70 (72%)	240 (59%)
Male N=338	269	118 (44%)	23	12 (52%)	28	14 (50%)	18	13 (72%)	157 (46%)
Transgender N=1	1	0 (0%)	0		0		0		0 (0%)

A Number reporting stigma (NRS) was a ‘yes’ or ‘no’ response to a question: Community members were asked whether they ‘Ever seen/heard community members with TB being stigmatized?’, while the 3 other study populations were asked whether they had ‘Ever experienced stigma?’.

### People with TB

Of 460 PWTB, 215 (47%) reported experiencing stigma. Stigma scores ranged from 0 to 41 of a possible maximum score of 48. The median (IQR) score was 22 (15–27); similar in those treated for MDR TB (median score 23, IQR 16–27) and those treated for drug-susceptible TB (22; 16–27). Table 2 lists findings from univariate and multivariate linear regression analyses to determine factors associated with self-stigma. Age and urban slum residence were independently associated. PWTB aged 65 and older reported an average self-stigma score 5.10 points lower (95% CI: -8.32, -1.89; p: 0.002) compared to PWTB younger than 65 years old, while those living in urban slums had higher average stigma scores (+3.89; 95% CI: 0.77–7.00; p: 0.015) than PWTB not living in urban slums. Stigma scores were lower in males than females, but the difference was not significant. Table 3 shows places where PWTB had ever experienced stigma and at what point along the care cascade. Of the health facilities, the hospital was the commonest site reported and the initial presentation with TB-related symptoms was the commonest time along the cascade. Stigma was also commonly reported to be experienced at home or at the workplace. In all settings, 135 (16.3%) reported experiencing stigma at either the end of treatment completion or at follow-up following treatment completion.

**Table 2.** Univariate and multivariate linear regression analysis of factors associated with experiencing stigma for PWTB.

Variable	Univariate analysis	P-value	Multivariate analysis	P-value
	Average change in self-stigma score: Unadjusted (95% CI)		Average change in self-stigma score: Adjusted (95% CI)	
Age (ref: 18-64 years; n=434)				
65 years or older (n=26)	-5.44 (-8.65–2.24)	0.001	-5.11 (-8.32–1.90)	0.002
Sex (ref: Female; n=190)				
Male (n=269)	-0.47 (-1.99–1.06)	0.548	-0.36 (-1.89–1.18)	0.649
Site of TB (ref: Extrapulmonary; n=162)				
Pulmonary (n=298)	1.10 (-0.46–2.67)	0.167	1.26 (-0.29–2.81)	0.112
Miner (ref: No; n=437)				
Yes (n=23)	-1.81 (-5.25–1.62)	0.300	-1.72 (-5.25-1.82)	0.340
Healthcare worker (ref: No; n=446)				
Yes (n=14)	1.40 (-2.96–5.76)	0.529	1.38 (-2.95–5.72)	0.530
Urban slum resident (ref: No; n=431)				
Yes (n=29)	4.22 (1.16–7.28)	0.007	3.88 (0.76–6.99)	0.015
Living in rural poor (ref: No; n=421)				
Yes (n=39)	2.35 (-0.34–5.03)	0.086	2.50 (-0.19–5.19)	0.068
Having a disability (ref: No; n=409)				
Yes (n=51)	0.95 (-1.43–3.34)	0.432	1.20 (-1.21–3.60)	0.328
Prisoner (ref: No; n =453)				
Yes (n = 7)	2.17 (-3.94-–.29)	0.486	2.13 (-4.06–8.33)	0.499

**Table 3.** Stigma experienced in specific settings by people with TB along the care journey by settings.

Care cascade	Hospital/clinic	PHC centre/ private clinic	Community/neighbours	Home/ family	Workplace	School	Prison	Number (%) of events by care cascade
	Number (%) of events of stigma experienced in each setting*							
Recognizing symptoms	59 (21.0%)	30 (20.7%)	25 (20%)	46 (32.4%)	36 (30.3%)	9 (60.0%)	0	205 (24.8%)
Seeking care	49 (17.4%)	31 (21.4%)	18 (14.4%)	9 (6.3%)	6 (5.0%)	1 (6.7%)	0	114 (13.8%)
Diagnosis evaluation	46 (17.1%)	37 (25.5%)	12 (9.6%)	11 (7.7%)	6 (5.0%)	1 (6.7%)	0	113 (13.6%)
Treatment initiation	30 (10.7%)	15 (10.3%)	25 (20%)	36 (25.4%)	27 (22.7%)	3 (20%)	1	137 (16.5%)
Treatment support	43 (15.3%)	18 (12.4%)	23 (18.4%)	17 (12%)	23 (19.3%)	0	0	124 (15.0%)
Completing treatment	18 (6.4%)	6 (4.1%)	10 (8%)	9 (6.3%)	9 (7.6%)	0	0	52 (6.3%)
Post-treatment follow-up	36 (12.8%)	8 (5.5%)	12 (9.6%)	14 (9.9%)	12 (10.1%)	1 (6.7%)	0	83 (10.0%)
Number (%) of events by sites	281 (33.9%)	145 (17.5%)	125 (15.1%)	142 (17.1%)	119 (14.4%)	15 (1.8%)	1 (0.1%)	828

* Note that some respondents reported experiencing stigma in more than one setting and at multiple steps along the care cascade.

### Family participants

Univariate linear regression analysis to evaluate the association between age, gender, relationship to the PWTB and the family secondary stigma score showed that the average variation in stigma scores was very small for all groups and that the confidence intervals all included zero. In the multivariate analysis, no significant variations were observed. Table 4 presents statements from the family secondary stigma scale and the number of respondents who agreed or strongly agreed with them. Of family member participants, 55 (61%) agreed or strongly agreed with the statement ‘I am worried about becoming infected’, and 54 (60%) agreed or strongly agreed with the statement ‘My family member hides his/her TB diagnosis from the community’. Only 16 (18%) reported that ‘I substitute another word for TB in the conversations with my friends’.

**Table 4.** Family secondary stigma scale statements and the number of people who agreed or strongly agreed with them.

Stigma perceptions	Number (%) of family members who agreed or strongly agreed with the family stigma scale statements
I am worried about becoming infected	55 (61%)
My family member hides his /her TB diagnosis from the community	54 (60%)
My family member asks to keep TB a secret	51 (57%)
I hide the fact that my family member has TB from the community	50 (56%)
I avoid talking about TB in the presence of other family members or neighbours	47 (52%)
I am afraid that someone will see me at the health care clinic where my relative is being treated	35 (39%)
I have noticed changes in my family member since the TB diagnosis	30 (33%)
I feel ashamed because my family member has TB	26 (29%)
I substitute another word for TB in the conversations with my family member	16 (18%)
I substitute another word for TB in the conversations with my friends	16 (18%)

### Community participants

Two-thirds of 83 community participants were female and 36 (43%) community members knew at least two PWTB personally. Overall, 93% of community members reported recognition of stigma against people affected by TB, commonly reporting observing stigma in hospitals and workplaces, particularly during periods where individuals were symptomatic. Table 1 reports that 51 (61%) of community members had ‘seen or heard of a member in your community who has TB being stigmatized because of his or her TB status’. An additional question was: ‘Do you know of community members with TB experiencing stigma in different settings that inhibited them from going to a DOTS centre, beginning treatment, adhering to treatment or completing treatment?’. The responses identified the prevalence and visibility of stigma within the community and its broader impact on treatment adherence and completion, highlighting that social stigma is a barrier to effective TB care.

### Healthcare workers

The stigma scores for HCWs ranged from 11 to 35 of a possible 0–36, with a median (IQR) of 25 (22–28). Results of univariate and multivariate linear regression analysis are shown in Table 5. Those providing care to people with TB and being a doctor had significantly higher median stigma scores compared to those not providing care. and compared to other HCWs respectively. After controlling for the effect of clinical role, providing service to people with TB, people aged 45 years or older had a median score 1.84 points higher (95% CI: 0.16–3.53, p: 0.033) than those younger than 45 years. Of 83 HCWs who reported stigma, 71 (86%) were stigmatised at the hospital and stigma was more commonly experienced in the community (n=21) than at home (n=16).

**Table 5.** Univariate and multivariate linear regression analysis of factors associated with healthcare worker stigma score.

Variable	Univariate analysis	P-value	Multivariate analysis	P-value
	Average change in stigma score (unadjusted) (95% CI)		Average change in stigma score (adjusted) (95% CI)	
Age				
45 years or older (n=55) ref: 18-44 years (n=60)	1.27 (-0.39-2.93)	0.133	1.84 (0.16-3.53)	0.033
Sex				
Male (n=18) ref: Female (n=97)	0.79 (-1.51-3.10)	0.495	0.27 (-2.22-2.77)	0.828
Clinical role				
Doctor (n = 54) ref: Other roles (n = 61)	2.07 (0.43-3.70)	0.014	2.10 (0.34-3.86)	0.020
Experience of providing service to people with or had TB				
Yes (n = 107) ref: No (n = 8)	4.19 (0.99-7.39)	0.011	3.63 (0.46-6.80)	0.025

## DISCUSSION

The findings from this first national TB stigma assessment in Mongolia provide a comprehensive view of the prevalent and multi-faceted nature of TB-related stigma in the country. Stigma is widespread and PWTB commonly reported that experienced stigma had negatively impacted their access to care, consistent with other studies identifying stigma as a factor in preventing timely care and compromising treatment outcomes.^14,15^ Once care was accessed, PWTB commonly reported experiencing stigma in clinical settings at treatment initiation. Stigma is associated with significant psychological morbidity leading to reduced treatment adherence and poorer treatment outcomes, and has been commonly reported from many settings as has the effect of social stigma and impact on families.^16–20^ National representative data can inform specific, locally relevant measures required to address stigma. While TB-related stigma is a common global phenomenon, there are a varied range of ethnic and cultural expressions with respect to knowledge of TB and its transmission as well as attitudes and health responses.^14^ The stigma scale first developed and validated by van Rie et al.^21^ has since been adapted, translated and validated in other populations.^22–24^ More recently, The Stop TB Partnership’s TB stigma assessment was developed using a multistakeholder approach ensuring community representation.^11^ This tool was utilised in our study as it provided guidance on broadening assessment beyond people with TB to others in the community who themselves are affected by TB or whose perceptions can impact on people with TB. Furthermore, the assessments were adapted from tools were previously developed and validated for relevant populations.^12,21,25,26^

A strength of this study was the inclusion of large number of PWTB currently or recently treated for TB, including MDR/RR TB, in multiple settings across Mongolia. Being resident in urban slums was associated with a higher stigma score, where TB prevalence is known to be higher in urban slums compared to those living in other urban areas.^2,5^ The possible reasons for stigma being less commonly reported by older people are not known but those aged 65 years and older represented less than 10% participants. Across all participant groups, females more commonly reported stigma experience but limited to PWTB this difference was not significant. Age and sex may affect perception and management including negative attitudes from healthcare providers.^14^

A major finding was the high prevalence of self-reported stigma in HCWs, highest in older HCWs. This may reflect generational differences in attitudes towards TB or workplace experiences. It is of particular concern that HCWs, especially doctors directly involved in TB care, commonly reported experiencing stigma themselves. This likely perpetuates negative attitudes toward PWTB potentially leading to suboptimal care^15^ as well as causing psychological stress and feelings of isolation in HCWs themselves in the workplace. Targeted stigma reduction training and support within the healthcare sectors is needed as well as education of HCWs throughout the care cycle.

Secondary stigma is commonly experienced by family members of PWTB. Over half of family members expressed concern about being infected and a similar proportion reported hiding the TB diagnosis from others. These behaviours likely reinforce social isolation of PWTB and their families. Furthermore, a high percentage of community members reported recognizing stigma against PWTB, particularly in healthcare settings and workplaces. Community-level interventions are necessary to reduce discrimination and promote more supportive environments for PWTB as well as to promote community-based interventions such as active case finding.^27^ Minimizing stigma is essential to TB prevention and care. Willingness to report may differ by characteristics such as age and sex. Our findings from observed experience and qualitative research have provided deeper insights into the specific experiences of stigma as well as identified issues of human rights and gender equity among key vulnerable populations to guide the development of more tailored interventions to reduce the prevalence and burden of stigma and will be reported separately. The contribution of community health workers in all 21 provinces, one-third of all 319 soums and eight districts of Ulaanbaatar, achieved a high participation rate and likely enhanced the study's reliability and generalizability. Collaboration with government programmes throughout aligned findings with national health priorities, facilitated policy implementation and precipitated nationwide human rights training and curriculum revisions under the Global Fund-supported TB project for 2024–2026. The Mongolian Anti-TB Coalition is also conducting public awareness campaigns and community engagement programs to reduce stigma, supported by the Stop TB Partnership's Civil Society Challenge Facility grant in 2024.

## CONCLUSION

This study highlights the pervasive nature of TB-related stigma in Mongolia and its significant impact on TB care and treatment. There is an urgent need for comprehensive stigma reduction strategies that focus beyond PWTB and their families to HCWs and the community.

## Supplementary Material


