# Supplementary material for: TB-related stigma is widely prevalent among people with TB and carers in Mongolia
Source: IJTLD Open. 2025 Jul 9;2(7):420–6. doi: 10.5588/ijtldopen.25.0174 (PMC12248404; doi:10.5588/ijtldopen.25.0174)
Supplement: Supplementary file 1 [file ijtldopen25-0174_supplementarydata1.pdf]

Supplementary file.

SAMPLE SIZE CALCULATION FOR PEOPLE WITH TB AND SAMPLING METHOD…………………………..2

Supplementary Table 1. Sampling of people with TB by all 21 provinces and 9 districts………….3

Supplementary Table 2. Comparison of key demographic and clinical characteristics between the study population and national data……………………………………………………………………………..5

CONSENT FORM 6

QUESTIONNAIRE 1: FOR PEOPLE WITH OR WHO HAVE HAD TB (AGED 18 OR OVER)................. 10

QUESTIONNAIRE 2: FOR FAMILY MEMBERS LIVING IN THE SAME HOUSE AS PEOPLE WITH OR WHO HAVE HAD TB (AGED 18 OR OVER)... 16

QUESTIONNAIRE 3: FOR COMMUNITY (RESIDENTS/NEIGHBOURS OF PEOPLE WITH OR WHO HAVE HAD TB, AGED 18 OR OVER).. 16

QUESTIONNAIRE 4: FOR HEALTHCARE WORKERS (HOSPITAL OR CLINIC STAFF WHO PROVIDE TB SERVtICES 18

Sample size calculation and random sampling

The sample size of individuals with TB was determined using the "TB Stigma Measurement Guidance" provided by the Netherlands TB Foundation (KNCV) which considers total number of people with TB and an assumed prevalence of stigma of 50%. The formula used to calculate sample size (n) was:

$$n = \frac{NP(1-P)}{(N-1)D^2 + P(1-P)}$$

N – The total number of people with TB (N=3000); P – Prevalence of TB stigma assumed (p=0.5); D – d/ t_α with d – limit of the stigma (d=5%) and t_α – 95% confidence interval (z=1.96).

Using this formula, the initial number of TB cases to be included was 341. After considering that 40% of patients might refuse to participate (as observed in a similar assessment conducted in Ukraine in 2019), the adjusted number of cases required for the study was 477, and so we aimed to recruit 480 people with TB.

Sampling

A simple random sampling method was used:

- A list of diagnosed TB cases was extracted from the electronic TB registration system (tubis.mn), including both drug-sensitive and drug-resistant cases, based on laboratory and clinical records.
- Duplicate entries were removed using registration numbers, names, age, and sex.
- Each individual was assigned a random number (using a random number function) from 1 to N in MS Excel.
- A separate column was generated using another random number function to create a list of values from 1 to N, and the first 480 cases were selected based on these random numbers (Table).

Supplementary Table 1. Sampling of people with TB by all 21 provinces and 9 districts

Provinces	Number of cases	Number of selected patients	%	Number of patients with MDR/RR-TB
Arkhangai	42	8	19.0%	3
Bayan-Ulgii	26	5	19.2%	2
Bayankhongor	46	7	15.2%	-
Bulgan	34	6	17.6%	3
Govi-Altai	22	3	13.6%	1
Govisumber	14	1	7.1%	-
Darkhan-Uul	123	17	13.8%	9
Dornogovi	60	9	15.0%	2
Dornod	84	8	9.5%	1
Dundgovi	22	4	18.2%	3
Zavkhan	21	3	14.3%	-
Orkhon	71	10	14.1%	3
Uvurkhangai	43	3	7.0%	2
Umnugovi	20	5	25.0%	1
Sukhbaatar	51	8	15.7%	2
Selenge	91	17	18.7%	8
Tuv	81	13	16.0%	1
Uvs	49	4	8.2%	-
Khovd	26	4	15.4%	1
Khuvsgul	71	18	25.4%	1
Khentii	87	12	13.8%	3
**Total in the provinces**	**1084**	**165**	**15.2%**	**46**
**Districts of Ulaanbaatar**				
Baganuur district	23	3	13.0%	-
Bagakhangai district	1	-	0.0%	1
Bayangol district	238	40	16.8%	12
Bayanzurkh district	456	63	13.8%	14
Nalaikh district	56	5	8.9%	-
Songinokhairkhan district	445	97	21.8%	32
Sukhbaatar district	131	27	20.6%	8
Khan-Uul district	181	34	18.8%	9
Chingeltei district	196	32	16.3%	9
Total in the city	1727	301	17.4%	85
Prison-429	68	11	16.2%	-

Enerel	21	3	14.3%	2
National Center for Mental Health	3	-	0.0%	1
**Total**	**2903**	**480**	**16.5%**	**134**

Supplementary Table 2. Comparison of key demographic and clinical characteristics between the study population and national data

Characteristics	National TB caseload	%	Study population	%	P-value*
**Age**					
Mean (SD)	39.1 (15.09)		38.8 (14.69)		0.42$^{\delta}$
**Gender**					
Male	1410	58.2%	267	58.0%	0.95
Female	1013	41.0%	193	42.0%	
Total	2423		460		
**Employment**					
Unemployed	721	29.8%	123	26.7%	0.06
Employed	721	29.8%	162	35.2%	
Other (pensioners, students etc.)	981	40.5%	175	38.0%	
Total	2423		460		
**Residence**					
Ulaanbaatar	1485	61.3%	294	63.9%	0.29
Provinces	938	38.7%	166	36.1%	
Total	2423		460		
**TB type**					
New	1598	86.2%	300	85.5%	0.73
Relapse	256	13.8%	51	14.5%	
Total	1854		351		
**Site of TB**					
Pulmonary TB	1343	69.7%	254	70.2%	0.90
Extra pulmonary TB	583	30.3%	108	29.8%	
Total	1926		362		

*Fisher's exact test
$^{\delta}$Independent t-test

CONSENT FORM

INTRODUCTION

Hello, my name is…….............……….... I work with ....…………….(NGO's name. It is an independent nongovernmental organization working with people affected by tuberculosis (TB), including people with or who have had TB, their families, neighbors in the community where they live, and healthcare workers who provide TB-related services.

Today, we are conducting a community study on TB-related stigma among (people with or who have had TB, family of people with or who have had TB, community/neighbors of people with or who have had TB, and healthcare workers) who are 18 years of age or older.

We would like to learn from you about your experience with TB-related stigma, as well as if you have heard or seen others being stigmatized. We are particularly interested in how stigma acts as a barrier to accessing TB services. We also welcome your input on how TB services, laws, and policies could be enhanced. Your responses will help to improve TB services in Mongolia.

This interview should take 30 minutes. You are free to leave the interview at any time without any consequences. You are also free to not answer any of the questions that you don't feel comfortable answering. Just let me know.

Your participation will not affect your or others' access to TB treatment. This survey is completely anonymous. No record of your name or any other personal information relating to you will be linked to your response. Interview notes will be analyzed only by the research team. Results of the survey will not disclose any information specific to you.

The research methodology has been approved by Scientific Committee of National Center for Communicable Diseases and Medical Ethics Review Committee of the Ministry of Health.

Do you have any questions? Do you have any objections to us proceed this interview?

We thank you for your time to help us with this important work. Before we start, I must ask you to state that you have understood everything that I just explained and that I answered all the questions you had. I would appreciate it if you could sign the consent form before we proceed with the interview.

As an incentive for taking the time to talk to us, we will provide you 10,000 MNT.

I, ... hereby confirm I am aged 18 or over and give my consent to participate in the TB Stigma Community Study interview being run by Mongolian anti-TB Coalition.

INTERVIEWEE'S DECLARATION OF INFORMED CONSENT:

1. I have been informed about the specified and additional purpose(s) for which my opinions will be collected, used, and disclosed, as described above.

2. I understand that my opinions may be used and disclosed for secondary purposes that are necessary to achieve the above-described specified purpose.

3. I voluntarily make this declaration and freely consent to the participation in the interview.

Signature or Fingerprints[1]:...

[1](If you are unable to sign)

Date: ....

Witness's name...

Signature of witness:...

Date:...

Name of the interviewer:……..

Signature of the interviewer:………..

Date:…...

Place of the interview:…..

** The informed consent will be made in 2 copies, one to be given to the participant and the other to be attached to the research documents.*

QUESTIONNAIRE 1: FOR PEOPLE WITH OR WHO HAVE HAD TB (AGED 18 OR OVER)

Interviewee No.	
Interview Location (city, province, soum, bagh, khoroo)	
Interview Date (Year/month/date)	
Interviewer's Initials	

A. I am going to read you a few statements that describe how you may feel about tuberculosis (TB). Please tell me on a 5-point scale how much you disagree or agree with each of the following statements, where:

0 – Strongly disagree
1 – Disagree
2 – Have no opinion
3 – Agree
4 – Strongly agree

If you don't want to respond to a statement or think the statement is not applicable to you, just let me know and we can skip to the next statement. ***[check only one box for each statement].***

	**Statements/Points**	**Strongly disagree**	**Disagree**	**Have no opinion**	**Agree**	**Strongly agree**
		**0**	**1**	**2**	**3**	**4**
A1	I feel hurt how others react to knowing I have TB.					
A2	I lose friends when I share with them that I have TB.					
A3	I feel alone.					
A4	I keep a distance from others to avoid spreading TB germs.					
A5	I am afraid to tell those outside my family that I have TB.					
A6	I am afraid of going to TB clinics because other people might see me there.					
A7	I am afraid to tell others that I have TB because they may think that I also have HIV/AIDS.					
A8	I feel guilty because my family has the burden of caring for me.					
A9	I choose carefully who I tell about having TB.					
A10	I feel guilty for getting TB because of my smoking, drinking, or other careless behaviors.					
A11	I am worried about having HIV/AIDS.					
A12	I am afraid to tell my family that I have TB.					

№	Question	Answer	X
**a1**	Do any of the above 12 statements also describe how you feel about TB yourself? **[check only one box below]**	Yes, at least one statement	
		No, none of the 12 statements **[Skip to Question B]**	
**a2**	Have any of these feelings you have about TB inhibited you from seeking and accessing TB services? **[check only one box below]**	Yes	
		No	

**B.**	Have you ever felt you were stigmatized because of your TB status? **[check only one box below]**	Yes	
		No **[Skip to Question E]**	
b1.	Have you experienced stigma in hospitals or clinics that inhibited you from continuing to seek and access TB services? **[check only one box below]**	Yes	
		No	
b2.	Have you experienced stigma from your neighbors in the community where you live that inhibited you from seeking and accessing TB services? **[check only one box below]**	Yes	
		No	
b3.	Have you experienced stigma at home that inhibited you from seeking and accessing TB services? **[check only one box below]**	Yes	
		No	
b4.	Have you experienced stigma at work that inhibited you from seeking and accessing TB services? **[check only one box below]**	Yes	
		No	

C. Please tell me more about when you experienced stigma. Have you experienced the stigma that inhibited you from seeking timely care, going to a DOTS facility, getting an accurate diagnosis, beginning treatment, adhering to treatment or completing treatment in hospitals/clinics, community/neighbors, home/family, workplace, school, or prison? ***[check all boxes that are applicable]***

Have you experienced stigma in these settings (right) that inhibited you from (below)…	C1	C2	C3	C4	C5	C6	C7
	Hospitals/ Clinics/ Dispensaries	Private Hospitals/ Clinics	Community / Neighbors	Home/ Family	Workplac e	School	Prison
Recognizing symptoms?							
Seeking care?							
Getting an accurate diagnosis?							
Beginning treatment?							
Getting treatment adherence support?							
Completing treatment?							
Getting post-treatment follow-up services?							
Others							

**D.**	Can you tell me more about the TB-related stigma you have experienced? (e.g.,: TB/HIV coinfection, public messages on TB, laws, and those laws and policies)? **[summarize the interviewee's key points in the space below]**

№	Асуулт	Хариулт	X
**E.**	Do you know of other people with or who have had TB being stigmatized because of their TB status? **[check only one box below]**	Yes	
		No	

**F.**	Do you know of other people with or who have had TB experiencing stigma in hospitals/clinics, community/neighbors, home/family, workplace, school, or prison that inhibited them from seeking timely care, going to a DOTS facility, getting an accurate diagnosis, beginning treatment, adhering to treatment or completing treatment? **[*check all boxes that are applicable*]**						
	F1	F2	F3	F4	F5	F6	F7
Have you experienced stigma in these settings (right) that inhibited you from (below)…	Hospitals/ Clinics/ Dispensaries	Private Hospitals/ Clinics	Communit y/ Neighbors	Home/ Family	Workpl ace	School	Prison
Recognizing symptoms?							
Seeking care?							
Getting an accurate diagnosis?							
Beginning treatment?							
Getting treatment adherence support?							
Completing treatment?							
Getting post-treatment follow-up services?							
Others							

**G.**	Can you tell me more about the TB-related stigma you have seen or heard of (e.g., somebody was refused access to services due to his/her TB status, a woman was divorced due to her TB status, or a family member with TB refused to disclose his/her status)? **[summarize the interviewee's key points in the space below]**

**3.**	What would you like to see changed in TB services, laws and policies to address TB-related stigma (e.g., in hospitals/clinics, community, family, workplace)? **[summarize the interviewee's key points in the space below]**
-Hospitals/clinics:	
-Community:	

- Family:
- Workplace:
-School:
-Prison:
-Other:

**I.**	Do you have further comments you would like to share with us? **[summarize the interviewee's key points in the space below]**

**J.**	How do you think tuberculosis care and service could be improved? **[summarize the interviewee's key points in the space below]**

№	Question	Answer	X
**K.**	What's your gender? **[check only one box below]**	Female	
		Male	
		Transgender	
		Other	

**L.**	How old are you? **[check only one box below]**	18-24	
		25-44	
		45-64	
		65 or older	

**M.**	Do you self-identify as any of the following? **[check only one box below]**	Person living with HIV	
		Miner	
		Health care worker	
		Urban slum dweller	
		Rural poor	

		Person who uses drugs	
		Person who has a disability	
		Indigenous person	
		Former prisoner	
		None	

**N.**	Which of the following statements is accurate about you? **[check only one box below]**	Completed TB treatment over one year ago	
		Completed TB treatment within the last year	
		Currently on TB treatment	
		Never had TB treatment	

**O.**	What type of TB were you last diagnosed with? **[check only one box below]**	Pulmonary TB	
		Multidrug-Resistant TB	
		Extensively Drug-Resistant TB	
		Drug-Resistant TB	
		Extrapulmonary TB	
		Don’t know	
**Thank you very much for your time today and for sharing so generously with us both your experiences and insights.**			

QUESTIONNAIRE 2: FOR FAMILY MEMBERS LIVING IN THE SAME HOUSE AS PEOPLE WITH OR WHO HAVE HAD TB (AGED 18 OR OVER).

Interviewee No.	
Interview Location (city, province, soum, bagh, khoroo)	
Interview Date (Year/month/date)	
Interviewer’s Initials	

A. I am going to read you a few statements that describe how you may feel about tuberculosis (TB). Please tell me on a 5-point scale how much you disagree or agree with each of the following statements, where:

0 – Strongly disagree
1 – Disagree
2 – Have no opinion
3 – Agree
4 – Strongly agree

If you don’t want to respond to a statement or think the statement is not applicable to you, just let me know and we can skip to the next statement. ***[check only one box for each statement].***

	Statements/Points	Strongly disagree	Disagree	Have no opinion	Agree	Strongly agree
		0	1	2	3	4
A1	My family member asks me to keep the TB a secret.					
A2	I feel ashamed because my family member has TB.					
A3	I hide the fact that my family member has TB from the community.					
A4	My family member hides his/her TB diagnosis from the community.					
A5	I avoid talking about TB in the presence of other family members or neighbors.					
A6	I'm afraid that someone will see me at the healthcare clinic where my relative is being treated.					
A7	I substitute another word for TB in conversations with my family members.					
A8	I substitute another word for TB in conversations with my friends.					
A9	I've noticed changes in my family members since the TB diagnosis.					
A10	I am worried about becoming infected					

№	Question	Answer	X
**B.**	Have you ever felt you were stigmatized because of your family member's TB status? **[check only one box below]**	Yes	
		No **[Skip to Question E]**	

**C.**	Have you experienced stigma in hospitals/clinics, community/neighbors, home/family, workplace, school, or prison that inhibited you from supporting your family member with TB to go to a DOTS centre, begin treatment, adhere to treatment, or complete treatment? **[*check all boxes that are applicable*]**

Have you experienced stigma in these settings (right) that inhibited you from (below)…	C1	C2	C3	C4	C5	C6	C7
	Hospitals/ Clinics/ Dispensaries	Private Hospitals /Clinics	Community/ Neighbors	Home/ Family	Workplace	School	Prison
Recognizing symptoms?							
Seeking care?							
Getting an accurate diagnosis?							
Beginning treatment?							
Getting treatment adherence support?							
Completing treatment?							

Getting post-treatment follow-up services?							
Others							

**D.**	Can you tell me more about the TB-related stigma you have experienced (e.g., being refused services by neighbourhood market vendors, being refused to attend community events) **[summarize the interviewee's key points in the space below]**

**№**	**Question**	**Answer**	**X**
**E.**	Have you seen or heard of other families being stigmatized because of their family member's TB status? **[check only one box below]**	Yes	
		No	

**F.**	Do you know of other families experiencing stigma in hospitals/clinics, community/neighbors, home/family, workplace, school, or prison that inhibited them from supporting their family members with TB to go to a DOTS center, begin treatment, adhere to treatment, or complete treatment? ***[check all boxes that are applicable]***						
Have you experienced stigma in these settings (right) that inhibited you from (below)…	E1<br>Hospitals/ Clinics/ Dispensaries	E2<br>Private Hospitals/ Clinics	E3<br>Community / Neighbors	E4<br>Home/ Family	E5<br>Workplace	E6<br>School	E7<br>Prison
Recognizing symptoms?							
Seeking care?							
Getting an accurate diagnosis?							
Beginning treatment?							
Getting treatment adherence support?							
Completing treatment?							
Getting post-treatment follow-up services?							
Others							

**G.**	Can you tell me more about the TB-related stigma you have seen or heard of (e.g., being refused services by neighborhood market vendors, being refused to attend community events)? **[summarize the interviewee's key points in the space below]**

**H.**	What would you like to see changed in TB services, laws and policies to address TB-related stigma (e.g., in hospitals/clinics, community, family, workplace)? **[summarize the interviewee's key points in the space below]**
Hospitals/clinics:	

Community:
Family:
Workplace:
School:
Prison:
Other:

**I.**	Do you have further comments you would like to share with us? **[summarize the interviewee's key points in the space below]**

**J.**	How do you think tuberculosis care and service could be improved? **[summarize the interviewee's key points in the space below]**

**№**	**Асуулт**	**Хариулт**	**X**
**K.**	What's your gender? **[check only one box below]**	Female	
		Male	
		Transgender	
		Other	

**L.**	How old are you? **[check only one box below]**	18-24	
		25-44	
		45-64	
		65 or older	

**M.**	How are you related to the person with TB in your family? **[check only one box below]**	Parent	
		Grandparent	
		Spouse	
		Partner	
		Child	
		Grandchild	
		Other relative **[please specify]**	

Thank you very much for your time today and for sharing so generously with us both your experiences and insights.

QUESTIONNAIRE 3: FOR COMMUNITY (RESIDENTS/NEIGHBOURS OF PEOPLE WITH OR WHO HAVE HAD TB, AGED 18 OR OVER)

Interviewee No.	
Interview Location (city, province, soum, bagh, khoroo)	
Interview Date (Year/month/date)	
Interviewer's Initials	

A. I am going to read you a few statements that describe how you may feel about tuberculosis (TB). Please tell me on a 5-point scale how much you disagree or agree with each of the following statements, where:

0 – Strongly disagree
1 – Disagree
2 – Have no opinion
3 – Agree
4 – Strongly agree

If you don't want to respond to a statement or think the statement is not applicable to you, just let me know and we can skip to the next statement. ***[check only one box for each statement].***

	**Statements/Points**	**Strongly disagree**	**Disagree**	**Have no opinion**	**Agree**	**Strongly agree**
		**0**	**1**	**2**	**3**	**4**
A1	Some people might not want to eat or drink with friends who have TB.					
A2	Some people feel uncomfortable being near those who have TB.					
A3	If a person has TB, some community members will behave differently towards that person for the rest of their life.					
A4	Some people do not want those with TB playing with their children.					
A5	Some people keep their distance from people with TB.					
A6	Some people think that people with TB are disgusting.					
A7	Some people do not want to talk to others with TB.					
A8	Some people are afraid of those with TB					
A9	Some people try not to touch others with TB					
A10	Some people may not want to eat or drink with relatives who have TB.					
A11	Some people prefer not to have those with TB living in their community.					

№	Question	Answer	X
B.	Have you seen or heard of a member in your community who has TB being stigmatized because of his or her TB status? **[check only one box below]**	Yes	
		No **[Skip to Question E]**	

C.	Do you know of community members with TB experiencing stigma in different settings (hospitals/clinics, community/neighbors, home/family, workplace, school, or prison) that inhibited them from going to a DOTS center, beginning treatment, adhering to treatment, or completing treatment? **[Таарах бүх хариултыг сонгоно уу]**

Do you know of community members with TB experiencing stigma in these settings (right) that inhibited them from (below)…	C1<br>Hospitals/ Clinics/ Dispensaries	C2<br>Private Hospitals/ Clinics	C3<br>Community / Neighbors	C4<br>Home/ Family	C5<br>Workplace	C6<br>School	C7<br>Prison
Recognizing symptoms?							
Seeking care?							
Getting an accurate diagnosis?							
Beginning treatment?							
Getting treatment adherence support?							
Completing treatment?							
Getting post-treatment follow-up services?							
Others							

D.	What stigma have you seen or heard of being experienced by a person with TB in your community? (e.g., somebody was refused access to services due to his/her TB status, a woman was divorced due to her TB status, or a family member with TB refused to disclose his/her status)? **[summarize the interviewee's key points in the space below]**

E.	What do you think the community can do to help address TB-related stigma? **[summarize the interviewee's key points in the space below]**

F.	Do you have further comments that you would like to share with us? **[summarize the interviewee's key points in the space below]**

G.	How do you think tuberculosis care and service could be improved? **[summarize the interviewee's key points in the space below]**

№	Question	Answer	X
**H.**	What's your gender? **[check only one box below]**	Female	
		Male	
		Transgender	
		Other	

**I.**	How old are you? **[check only one box below]**	18-24	
		25-44	
		45-64	
		65 or older	

**J.**	How many people with or who have had TB in your community do you know personally? **[check only one box below]**	None	
		One only	
		Two or more	
		Don't know	

Thank you very much for your time today and for sharing so generously with us both your experiences and insights.

QUESTIONNAIRE 4: FOR HEALTHCARE WORKERS (HOSPITAL OR CLINIC STAFF WHO PROVIDE TB SERV

Interviewee No.	
Interview Location (city, province, soum, bagh, khoroo)	
Interview Date (Year/month/date)	
Interviewer's Initials	

A. I am going to read you a few statements that describe how you may feel about tuberculosis (TB). Please tell me on a 5-point scale how much you disagree or agree with each of the following statements, where:

0 – Strongly disagree
1 – Disagree
2 – Have no opinion
3 – Agree
4 – Strongly agree

If you don't want to respond to a statement or think the statement is not applicable to you, just let me know and we can skip to the next statement. ***[check only one box for each statement].***

	Statements/Points	Strongly disagree	Disagree	Have no opinion	Agree	Strongly agree
		0	1	2	3	4
A1	Some health care workers are nervous about treating TB patients.					
A2	Some health care workers feel pity for TB patients.					

A3	Some health care workers don't like helping TB patients.					
A4	Some health care workers stay away from TB patients.					
A5	Some health care workers think developing TB is the person's own fault					
A6	Some health care workers feel angry towards TB patients.					
A7	Some health care workers think it would be best for TB patients to be isolated during the intensive phase of treatment.					
A8	Some health care workers feel TB patients are dangerous.					
A9	Some health care workers think taking TB treatment should be forced if necessary.					
A10	Some health workers are hesitant to say they work in a TB hospital/clinic/dispensary.					

**№**	**Question**	**Answer**	**X**
**B.**	Have you ever felt you were stigmatized because your work involves interacting with people with or who have had TB? **[check only one box below]**	Yes	
		No **[Skip to Question E]**	

**C.**	Have you experienced stigma in hospitals/clinics where you work, from the community/neighbours where you live, and from your family/relatives? **[check all boxes that are applicable]**	Hospital/clinic/dispensary where you work	
		Community where you live	
		Your family/relatives	

**D.**	Can you tell me more about the TB-related stigma that you have experienced? (e.g., being refused se vices by neighbourhood market vendors due to your work with people with TB, not being invited to family gatherings due to your work with people with TB, being refused to attend religious gatherings) **[summarize the interviewee's key points in the space below]**

**№**	**Question**	**Answer**	**X**
**E.**	Have you seen or heard of your fellow health care workers being stigmatized because their work involves interacting with people with or who have had TB? **[check only one box below]**	Yes	
		No **[Skip to Question H]**	
**F.**	Do you know of any of your fellow health care workers being stigmatized in hospitals/clinics where they work, by community/neighbours where they live or by their family/relatives? **[check all boxes that are applicable]**	Hospital/clinic/dispensary where you work	
		Community where you live	
		Your family/relatives	

**G.**	Can you tell me more about TB-related stigma experienced by fellow health workers (e.g., being refused services by neighbourhood market vendors due to their work with people with TB, not being invited to family gatherings due to their work with people with TB, being refused to attend religious gatherings) **[summarize the interviewee's key points in the space below]**

**H.**	What do you think health care workers can do to help address TB-related stigma? **[summarize the interviewee's key points in the space below]**

**I.**	Do you have further comments that you would like to share with us? How do you think tuberculosis care and service could be improved? **[summarize the interviewee's key points in the space below]**

**№**	**Question**	**Answer**	**X**
**J.**	What's your gender? **[check only one box below]**	Female	
		Male	
		Transgender	
		Other	

**K.**	How old are you? **[check only one box below]**	18-24	
		25-44	
		45-64	
		65 or older	

**L.**	What is your position?? **[check only one box]**	Doctor	
		Nurse	
		Other **[Please specify]**	

**M.**	Have you provided services to people with TB before? **[check only one box]**	Yes	
		No	

**N.**	Have you attended any human rights-related training in the last year?? **[check only one box]**	Yes	
		No	

**O.**	Have you attended any training related to discrimination in the last 1 year? **[check only one box]**	Yes	
		No	

**P.**		Yes	

	Have you received any human rights training before the Covid-19 pandemic or before 2019?? **[check only one box]**	If yes, when and which training?..	
		No	

**Q.**	Have you received any training on discrimination before the Covid-19 pandemic or before 2019?? **[check only one box]**	Yes	
		If yes, when and which training?..	
		No	

**R.**	Were the costs of the human rights and anti-discrimination training you attended included in your organization's budget? **[check only one box]**	Yes	
		No	
		Don’t know	

Thank you very much for your time today and for sharing so generously with us both your experiences and insights.
